# The association of hormone signalling genes, transcription and changes in shoot anatomy during moso bamboo growth

**DOI:** 10.1111/pbi.12750

**Published:** 2017-06-09

**Authors:** Long Li, Zhanchao Cheng, Yanjun Ma, Qingsong Bai, Xiangyu Li, Zhihua Cao, Zhongneng Wu, Jian Gao

**Affiliations:** ^1^ International Center for Bamboo and Rattan Key Laboratory of Bamboo and Rattan Science and Technology State Forestry Administration Beijing China; ^2^ Anhui Academy of Forestry Hefei Anhui Province China

**Keywords:** shoot anatomy, endogenous hormone, fast growth, moso bamboo, hormone signalling genes

## Abstract

Moso bamboo is a large, woody bamboo with the highest ecological, economic and cultural value of all the bamboo types and accounts for up to 70% of the total area of bamboo grown. However, the spatiotemporal variation role of moso bamboo shoot during growth period is still unclear. We found that the bamboo shoot growth can be divided into three distinct periods, including winter growth, early growth and late growth based on gene expression and anatomy. In the early growth period, lateral buds germinated from the top of the bamboo joint in the shoot tip. Intercalary meristems grew vigorously during the winter growth period and early growth period, but in the late growth period, mitosis in the intercalary meristems decreased. The expression of cell cycle‐associated genes and the quantity of differentially expressed genes were higher in early growth than those in late growth, appearing to be influenced by hormonal concentrations. Gene expression analysis indicates that hormone signalling genes play key roles in shoot growth, while auxin signalling genes play a central role. *In situ* hybridization analyses illustrate how auxin signalling genes regulate apical dominance, meristem maintenance and lateral bud development. Our study provides a vivid picture of the dynamic changes in anatomy and gene expression during shoot growth in moso bamboo, and how hormone signalling‐associated genes participate in moso bamboo shoot growth.

## Introduction

Moso bamboo (*Phyllostachys edulis*) is a lignocellulose‐abundant plant with great ecological, economic and cultural value. It is part of the monophyletic BEP clade (Bambusoideae, Ehrhartoideae, Pooideae) in the grass family (Poaceae) and has one of the fastest growth rates among all plants (Lin *et al*., 2010). In suitable spring conditions, the shoot can grow as fast as 1 m/day at peak growth and reach heights of 20 m in 45–60 days. Due to the amazing growth rate and unique strength, moso bamboo generates an equivalent of 5 billion US dollars including both timber and nourishment (Peng *et al*., 2013a).

To better understand the nature of bamboo's unique growth, previous studies have finely characterized bamboo shoot growth and anatomy (Lee and Chin, 1960; Lin *et al*., 2002). Of greatest importance is the mediacy meristem, which vigorously divides and promotes growth through continuous longitudinal divisions. To build on this understanding, the anatomical and physiological foundations during this life cycle need to be studied.

Recently, several genomic studies of moso bamboo have been conducted, including cDNA sequencing (Peng *et al*., 2010), ESTs (Zhou *et al*., 2011), internode elongation‐associated protein expression analysis (Cui *et al*., 2012) and alternative splicing (Li *et al*., 2016). Furthermore, the recent publication of the draft genome sequence (Peng *et al*., 2013b) led to an understanding of molecular mechanisms during shoot growth. Digital expression profiles of four different floral stages of moso bamboo identified highly expressed genes involved in stress‐responsive and GA‐signalling pathways indicating a potential connection between adversity stress, GA and bamboo flowering (Gao *et al*., 2014). Previous transcriptomic sequencing analyses have revealed that a plant hormonal network, cell cycle regulation and cell wall metabolism can regulate fast shoot growth in moso bamboo (He *et al*., 2013; Peng *et al*., 2013a). However, these previous studies were limited by composite samples and the absence of a reference genome leading to a relative undercharacterization of moso bamboo shoot compared other model plants. Rice (Luo *et al*., 2006 and Zhou *et al*., 2006), tobacco (Zhao *et al*., 2013) and *Populus* (Ruonala *et al*., 2008) have genes involved in stem or internode elongation that are well defined, while this information is lacking in bamboo.

In the current research, we divided bamboo growth into three distinct growth periods including winter growth (underground period), early growth (0–8 m in height) and late growth (8–12 m in height), based on the gene expression and anatomy. Shoots from eight different growth stages were collected including winter bamboo shoot, 50, 100, 300, 600, 900, 1200 cm and culm, after leaf expansion. The tissues above the fifth bamboo joint from the shoot tip were isolated and sequenced on Illumina HiSeq™ 2000 to comprehensively characterize the molecular basis of physiological processes during the whole shoot growth period. We also explored the spatiotemporal variation of moso bamboo shoots during growth. Our results will lay a foundation for further gene expression and functional genomic studies in bamboo.

## Results

### Daily height measurement of bamboo shoots

**Figure 1:**
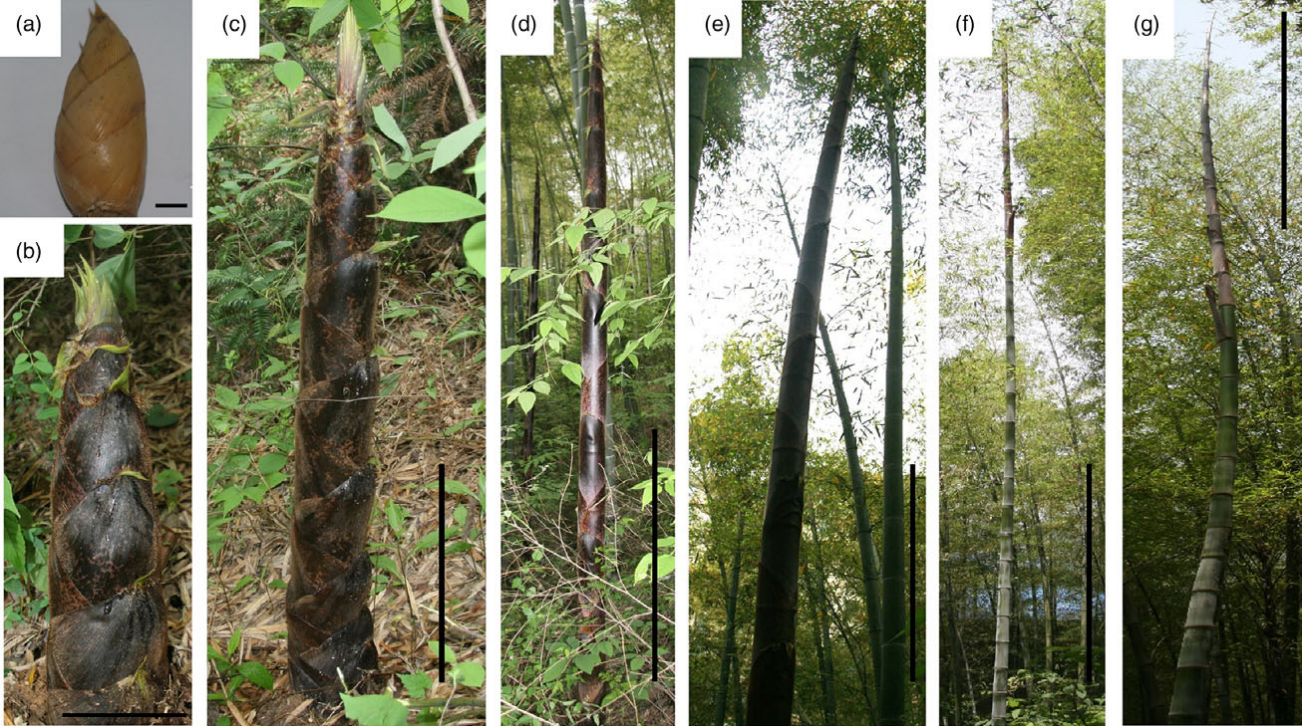
Examples of moso bamboo shoots in seven growth stages (a–g represented for S1–S7). Bars, 1 cm (a); 15 cm (b); 30 cm (c); 1 m (d); 1.5 m (e); 3 m (f); and 4 m (g).

**Figure 2:**
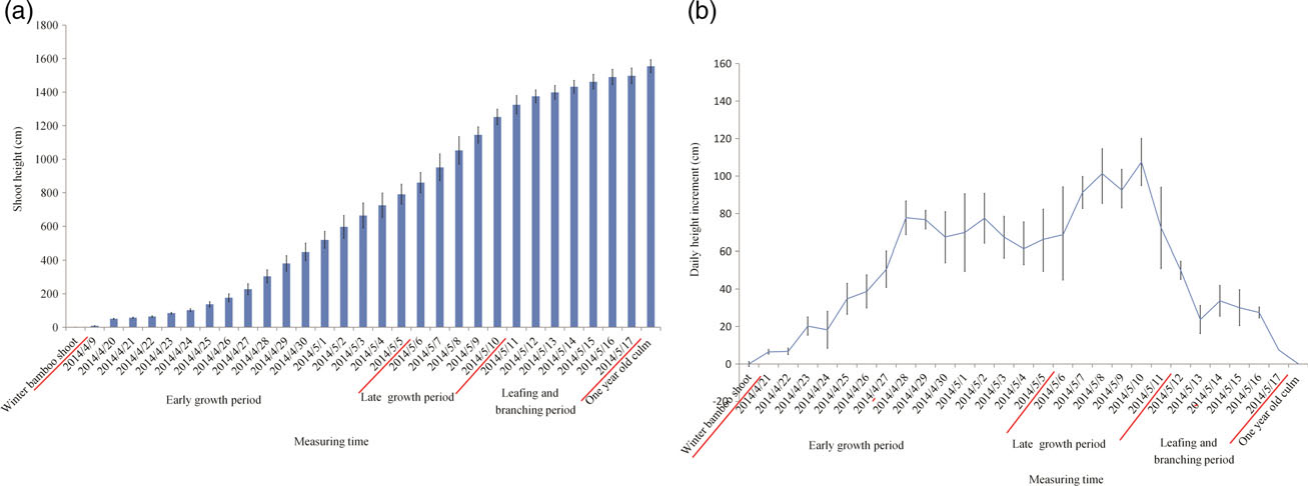
Daily height variation (a) and daily height increments of bamboo shoots (b). The horizontal axis represents the survey date, and vertical axis represents the shoot height (a) and height increment per day (b), respectively.

The heights of five individual moso bamboo shoots were measured at 8 A.M. every morning from April 2nd to June 20th to catalog height increments for the entirety of growth (Figure 1). The growth rates followed a bell curve (Figure 2a) with a low growth rates seen early (April 9th to April 24th). After reaching an average height of 102.8 cm on April 24th, daily shoot height gradually increased to the first growth peak (77.8 cm/day), achieving an average height of 307.0 cm (April 28th). Then a high rate of growth was maintained at 61.3–77.8 cm per day (April 28th to May 5th; Figure 2b). In the late growth period, the daily shoot height increment was much longer than that in early growth period. Finally, the shoot tips emerged from the shoot sheath and began to branch and leaf on May 12th (1375 cm), indicating an end of the shoot growth. Based on the continuing measurement of bamboo shoot height, we selected eight representative growth stages (winter bamboo shoot, 50, 100, 300, 600, 900 and 1200 cm) for anatomical changes, hormone content and transcriptome analysis.

### Dynamic anatomical variation in shoot tip growth stages

**Figure 3:**
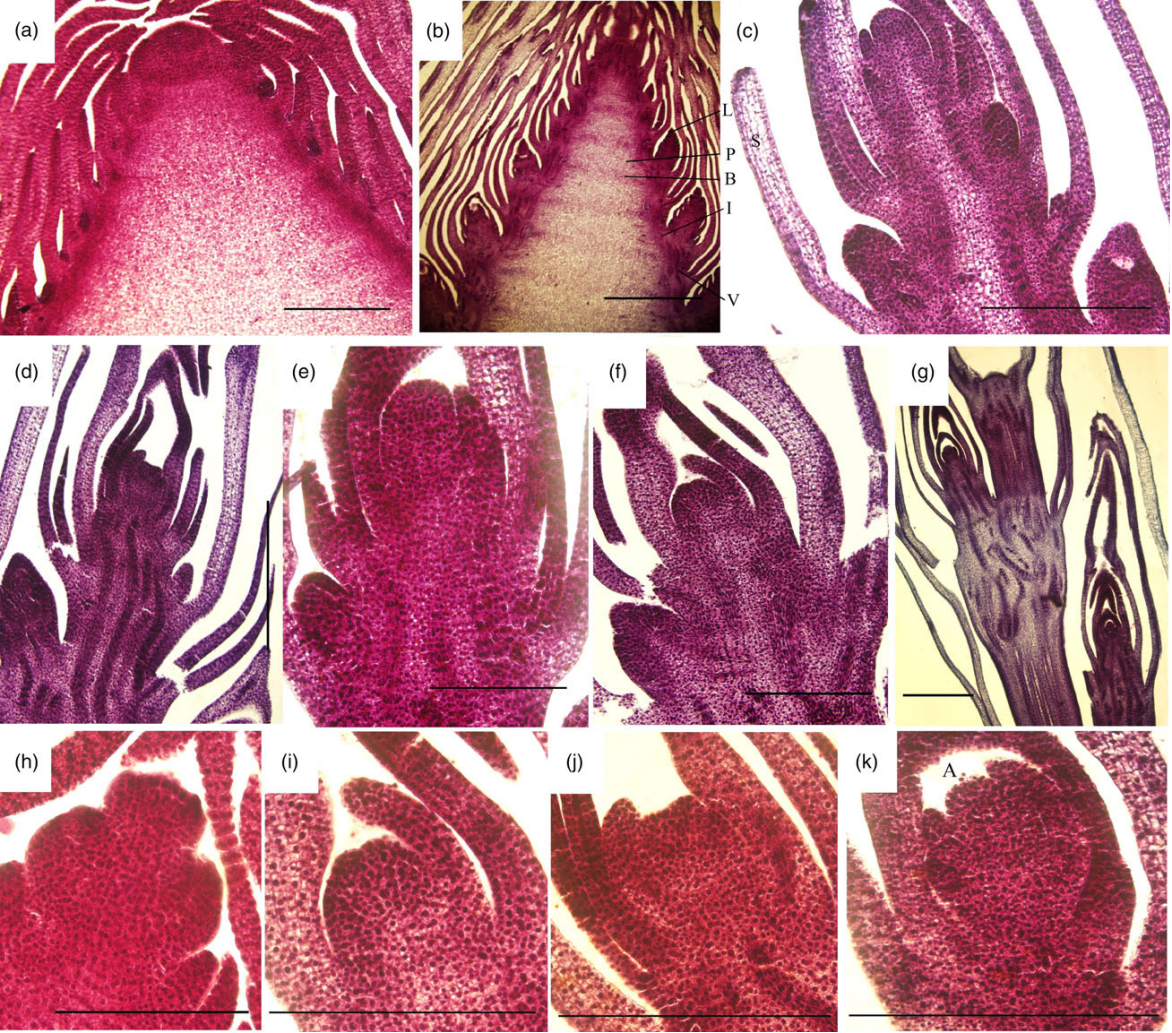
Transverse section of moso bamboo shoot tips in seven growth stages (S1–S7). (h–k) Apical meristems of S1, S3, S4 and S6. Bars, 2 mm (a–g); 500 μm (h–k). L is lateral bud, I is internode, B is bamboo joint, A is apical bud, V is vascular bundle, S is bamboo sheath and P is pith.

**Figure 4:**
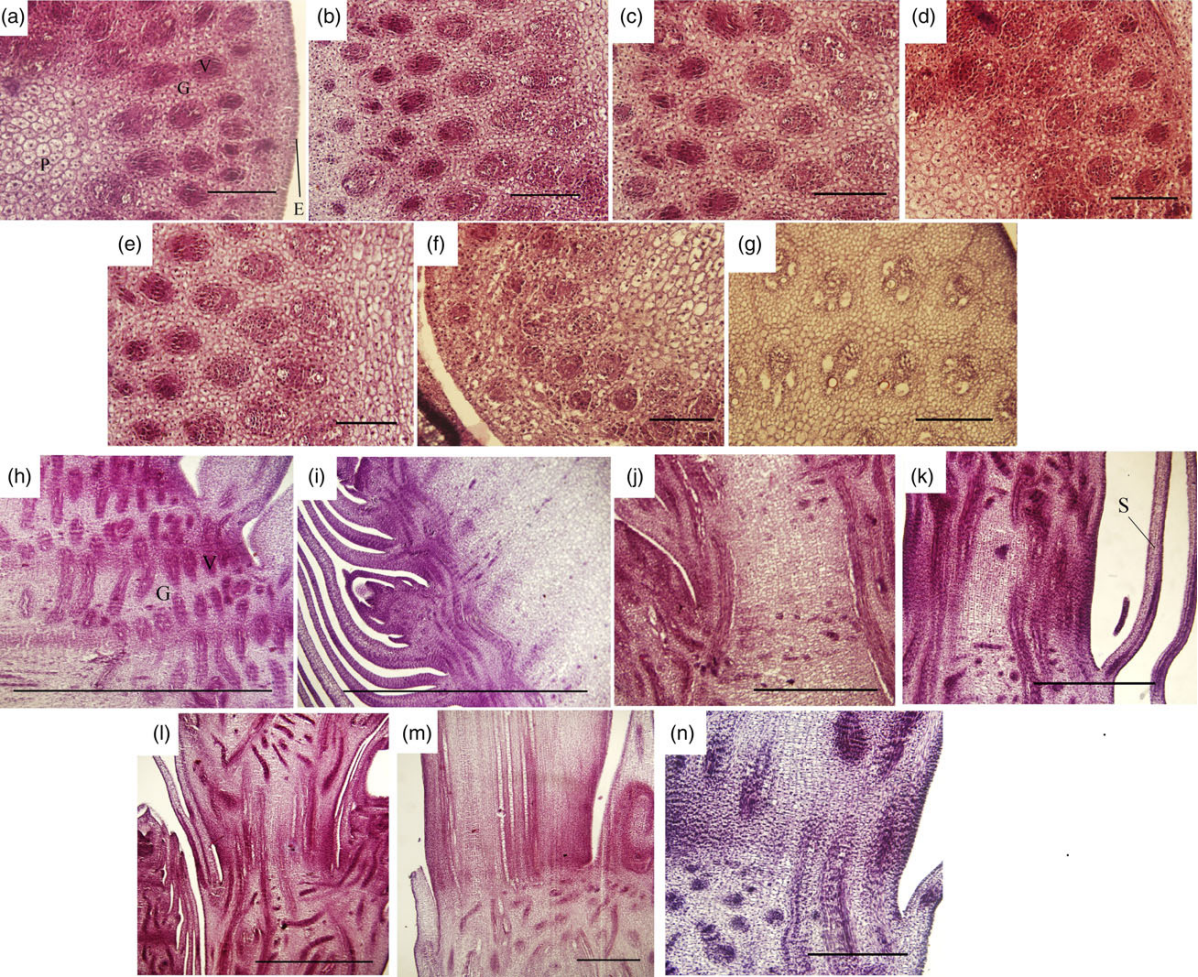
Cross‐sections and longitudinal sections of tissue from developing shoots (the fifth internode) in seven growth stages (S1–S7). (a–g) Cross‐sections of the fifth internode. Bars, 200 μm. (h–n) Longitudinal sections of the fifth internode. Bars, 500 μm. E, G, P, S and V represent epidermis, ground tissues, pith, bamboo sheath and vascular bundles, respectively.

**Figure 5:**
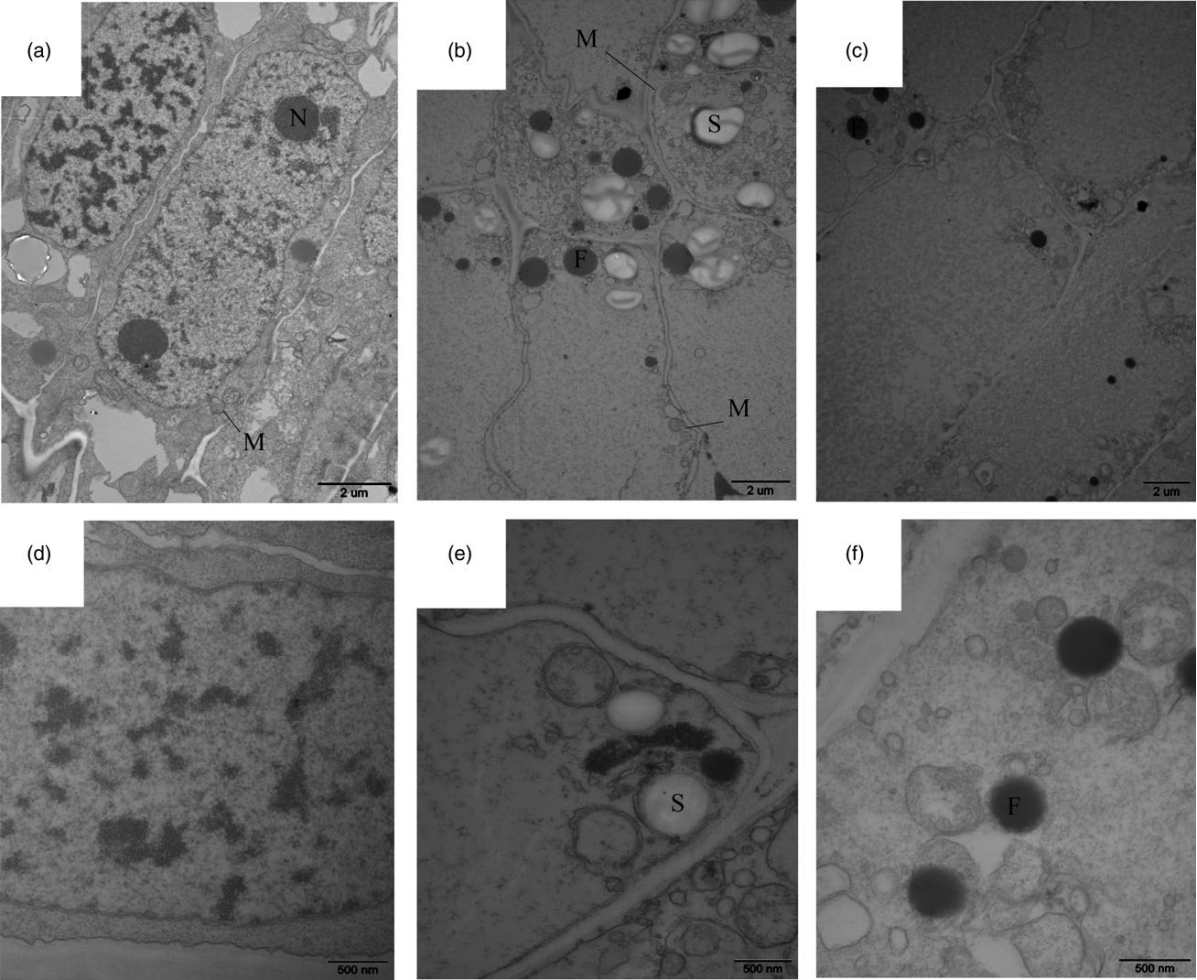
Internal structure of moso bamboo shoot cells (a, d) S1 stage, (b, e) S4 stage, (c, f) S6 stage. The N, M, F and S represent nucleolus, mitochondria, fat droplets and starch grains, respectively.

During shoot growth, the apical meristem has an exuberant division ability and can be divided into vegetative cone and subapical meristem (Figures 3a and S1). At S1, the shoot tip appeared as a pyramid without lateral buds (Figure 3a). As the bamboo shoots grew, shoot tips gradually bifurcated, and lateral buds at the top of bamboo joint were observed from S2 to S7 (Figure 3b–g). In the early growth period (S1 to S5), numerous cell nuclei were detected in ground tissue and vascular tissue, indicating the presence of meristematic tissue (Figure 4a–e). With the shoot growing, the number of nuclei declined until only a minority could be detected in the S7 (Figure 4f–g). Compared with cells in S1, the cells in S4 and S6 were much larger and had many more fat droplets (Figure 5). The endoplasmic reticulum and mitochondria were present on the edges of cells in S4 and S6 stages, due to the presence of a large central vacuole (Figure 5b,c). In summary, unlike the continually active apical meristem, cell division rates declined in intercalary meristems in the late growth period.

### Temporal and spatial variation of endogenous hormones during different developmental stages

**Figure 6:**
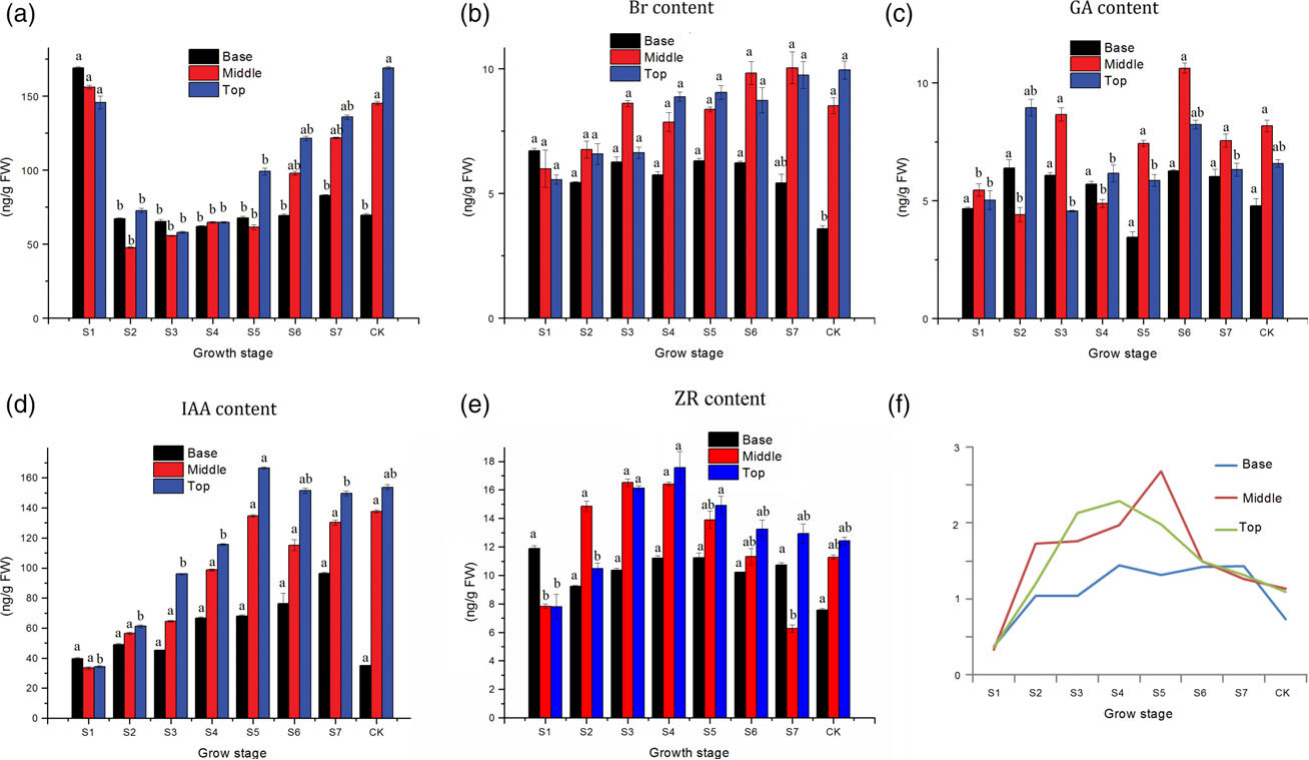
Dynamic changes in the concentration of five endogenous hormones in moso bamboo shoots at different developmental stages and internodes: abscisic acid (ABA) (a), brassinolide (Br) (b), indole acetic acid (IAA) (c), gibberellic acid (GA_3_) (d) and zeatin riboside (ZR) (e). Different letters on a column with the same pattern indicate significant differences at *P* ≤ 0.05 according to an LSD test. In a–e, the horizontal axis represents the growth stage, and the vertical axis represents the endogenous hormone content (ng/FW). (f) The ratio between ZR, BR, GA_3_ and IAA and ABA at different growth stages.

To better understand the roles of endogenous hormones involved in moso bamboo shoot growth, the concentration of five endogenous hormones, IAA, GA_3_, BR, ZR and ABA was measured using ELISA. In S1 (winter bamboo shoot), ABA was highly concentrated in B (basal parts), but decreased after S1. In contrast, the ABA content in both the M (middle part) and T (top part) decreased first, but increased steadily from S2 to CK. The concentration of BR content in B remained stable from S1 to S6 and then decreased. In contrast, the BR content increased in the M and T of the shoots displayed (Figure 6b). The temporal changes in the GA_3_ concentrations for all three parts (B, M, and T) were generally bimodal in nature. The first peak of B, M and T appeared at S2, S3 and S2, respectively, and the second peaks of the three parts all appeared in S6 (Figure 6c). In the T and M, IAA content in the shoots increased steadily from S1 to S5 and maintained a high concentration from S5 to CK. The IAA content in B increased slowly from S1 to S7 and decreased thereafter (Figure 6d). The dynamic change of ZR concentrations in the T was unimodal with a peak at S4 (Figure 6e). The ZR content in the M increased to peak at S4 and then decreased until another peak appeared at the CK stage. ZR content in the B had a bell‐curved profile. In addition, the ratio between growth promoting factors (ZR, BR, GA_3_, and IAA) and the inhibitory factor (ABA) was calculated across eight growth stages. The highest ratio of B, M and T appeared at S7, S5 and S4 stages, respectively. With the trend of ratio change stayed consistent with cell division ability of intercalary meristem, enticing an investigation of the molecular processes at the transcriptome level.

### Overview of gene expression at different shoot growth stages

To identify the key genes involved in shoot elongation, we performed paired‐end transcriptome sequencing of shoots from seven different growth stages using the Illumina HiSeq™ 2000. 81.10% to 85.2% of reads from each library mapped perfectly to the reference genome (Table S2; Figure S2).

**Figure 7:**
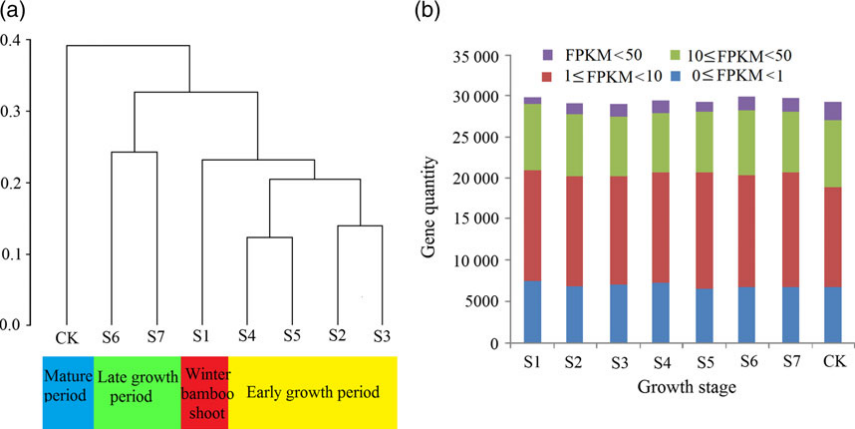
(a) Transcriptome relationships among different samples. (b) Expression of genes in eight samples. The *x*‐axis and *y*‐axis in each chart represent gene quantity and the eight growth stages, respectively.

The expression differences at different shoot growth stages resulted in two primary clusters, excluding the culm (CK) (Figure 7a). Shoot samples from S1 to S5 clustered together, corresponding to the winter period and early period of shoot growth, although S1 (winter shoot) was distant from the others. The S6 to S7 formed another cluster, representing the late period of bamboo shoot growth. The shoots showed different growth characteristics in two clusters, with accelerated growth in the early period, but kept a rapid growth rate per day in late period. The clustering result certified the reliability of the period division based on anatomical structure. The expression of genes between 1 FPKM and 10 FPKM accounted for the largest proportion in all shoot samples (Figure 7b). The genes with the highest expression (FPKM ≥50) continued to increase through the whole shoot growth period.

**Figure 8:**
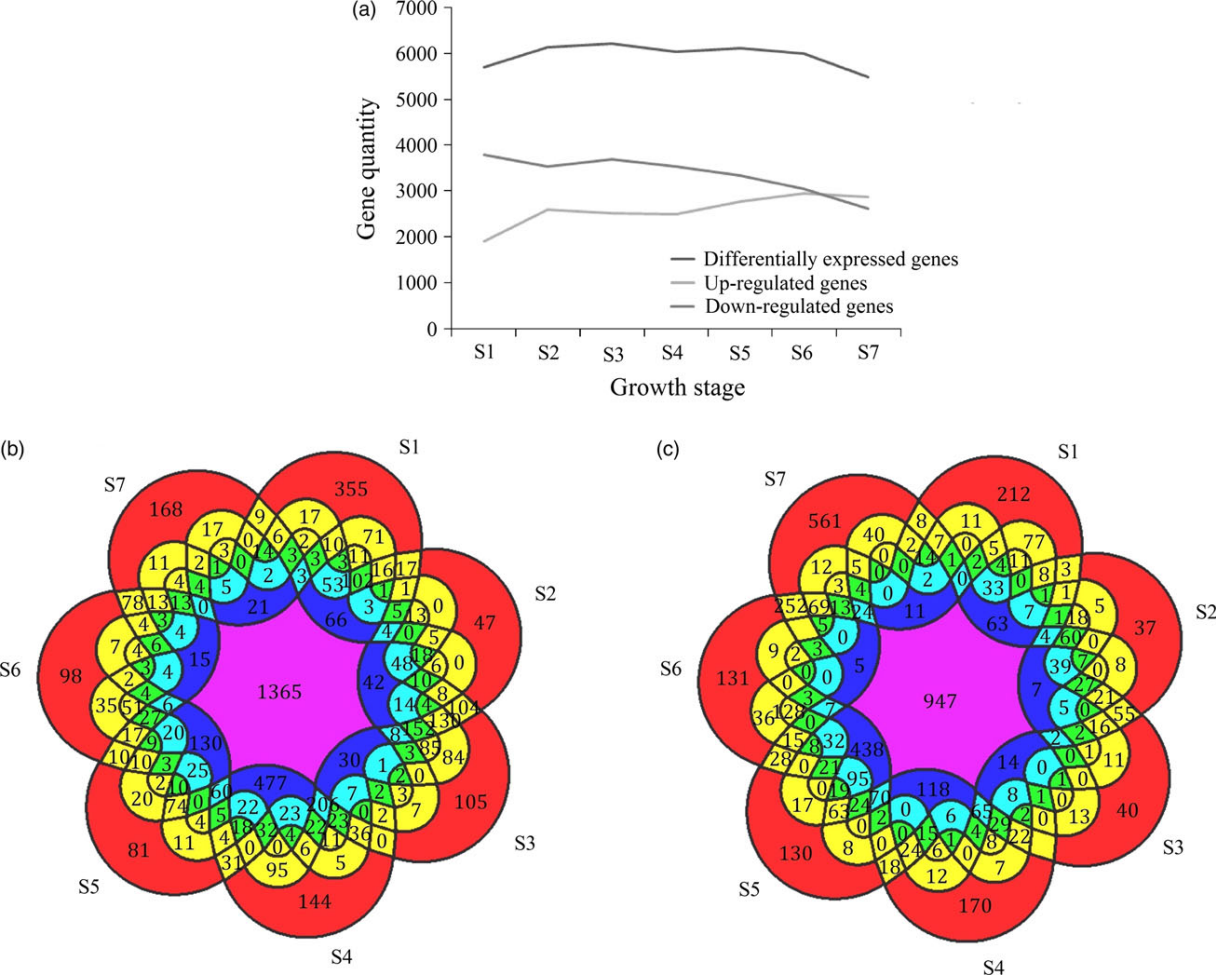
Differentially expressed genes related to shoot elongation. (a) Statistical graph of differentially expressed transcripts at seven developmental stages. (b) Overlapping sets of induced transcripts in seven different growth stages. (c) Overlapping sets of repressed transcripts in seven different growth stages.

By comparing all samples to CK, a total of 10 344 genes were differentially expressed during shoot growth, in at least one growth stage (Figure S3). In detail, the number of up‐regulated genes gradually increased to a high of 3089 in S7. In the early stages (S1 to S4), the number of down‐regulated genes fluctuated from 3544 to 3787 and declined after S4 (Figure 8a). An analysis of up‐ and down‐regulated DGEs shared between the different growth stages indicated that two adjacent stages shared more DGEs than two nonadjacent stages. For example, the number of DGEs shared by S2 and S3 (including 2360 up‐regulated genes and 3245 down‐regulated genes) was much higher than those shared by S2 and S6 (including 2083 up‐regulated genes and 2472 down‐regulated genes; Figure 8b,c). Only 1365 genes were simultaneously up‐regulated from S1 to S7, and only 947 genes were simultaneously down‐regulated from S1 to S7. In short, the quantity of DGEs was much higher in the early growth period, compared to the late growth period. Similarly, the intercalary meristem also had an exuberant ability to divide in the early growth period, but declined in later growth period.

### Quantitative real‐time RT‐PCR (qRT‐PCR) validation of gene expression

**Figure 9:**
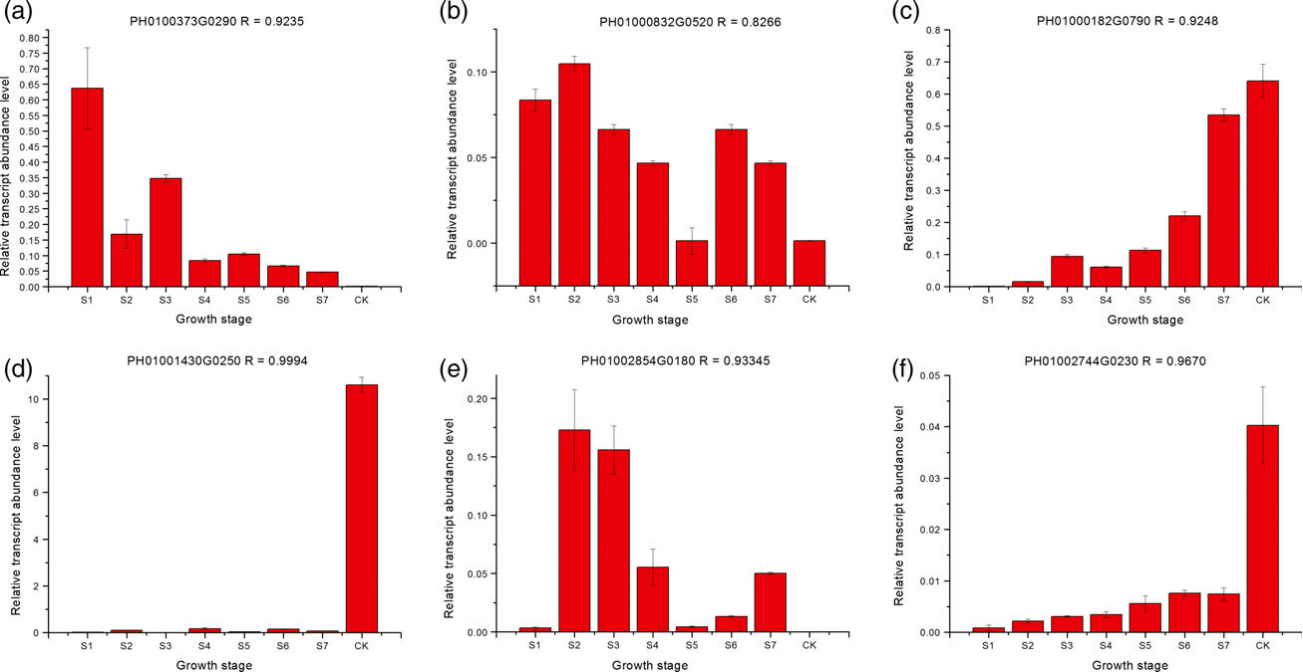
Verification of DGEs by qRT‐PCR. The relative amount of mRNA (*y*‐axis) is a ratio normalized by *TIP41* (tonoplast intrinsic protein 41). The shoot growth stages are on the *x*‐axis. R indicates the correlation coefficient for the expression between RNA‐Seq and qRT‐PCR data. The expression of each gene in CK was arbitrarily set at 1.0.

To validate the DGEs identified in the RNA‐seq, qRT‐PCR was performed on six genes. As expected, the qRT‐PCR confirmed the expression trends established in the DGE analysis with RNA‐seq. The qRT‐PCR data and transcriptome data were in close agreement indicating that the transcriptome results were highly reliable with Pearson correlation coefficients ranging from 0.827 to 0.999 (Figure 9).

### Functional analysis of DGEs

**Figure 10:**
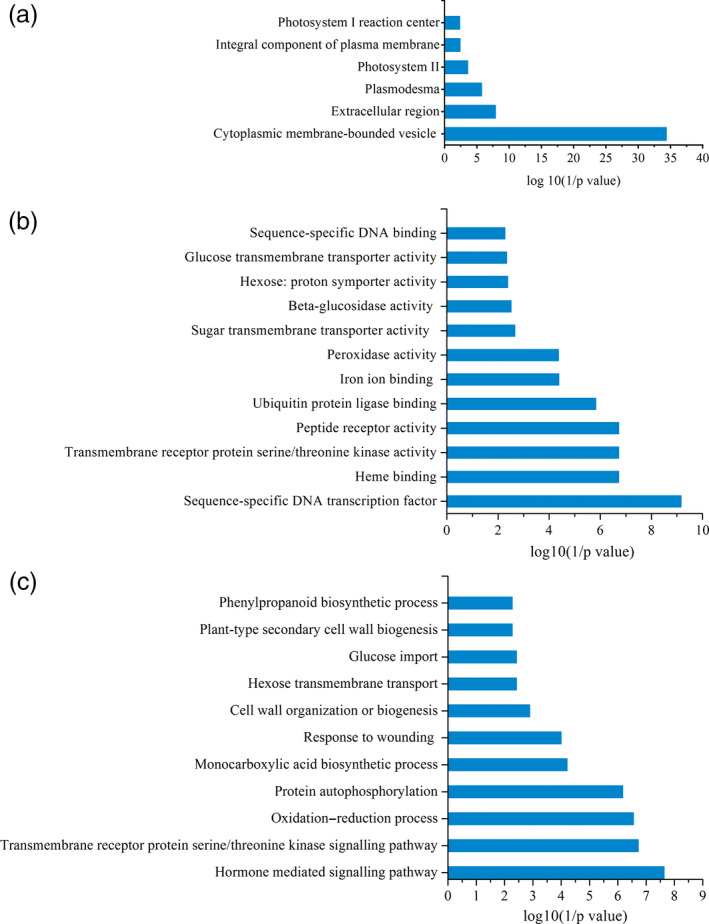
GO terms that were significantly enriched (FDR <0.05) in the DGEs (a) GO component, (b) GO function, (c) GO biological process.

To gain further insight into the changing transcriptomic landscape during shoot elongation, we performed an enrichment analysis gene ontology (GO) annotations. Based on sequence homology, 10 344 genes were categorized into the three main GO categories including biological process, cellular component and molecular function (Figure 10). In the cellular component category, ‘the cytoplasmic membrane‐bounded vesicle’, ‘extracellular region’ and ‘plasmodesmata’ accounted for the top three DGE categories. In the molecular function category, the greatest number of DGEs fell in the categories of ‘sequence‐specific DNA binding transcription factor activity’, ‘heme binding’ and ‘transmembrane receptor protein serine/threonine kinase activity’. Within the biological category, the groups with the highest abundance of DGEs included ‘hormone‐mediated signalling pathway’, ‘transmembrane receptor protein serine/threonine kinase signalling pathway’ and ‘oxidation–reduction process’, although other interesting groups included ‘protein autophosphorylation’ and ‘monocarboxylic acid biosynthetic process’ (Figure 10).

### Expression analysis of cell cycle‐associated genes

The E2F family, NAC family and the TGA family had more up‐regulated genes than down‐regulated (Tables S3 and S4). The number of up‐regulated *cyclin D* genes increased slowly from S1 to S3, reaching the highest at S3 and decreasing thereafter. The number of up‐regulated genes in *cyclin A*,* cyclin B* and *cell division cycle* was lowest at S1 and remained at high levels from S2 to S7 (Tables S3 and S4). Generally speaking, most cell cycle‐associated genes were up‐regulated during moso bamboo shoot growth, when compared with CK.

### Expression analysis of hormone signalling‐associated genes

**Figure 11:**
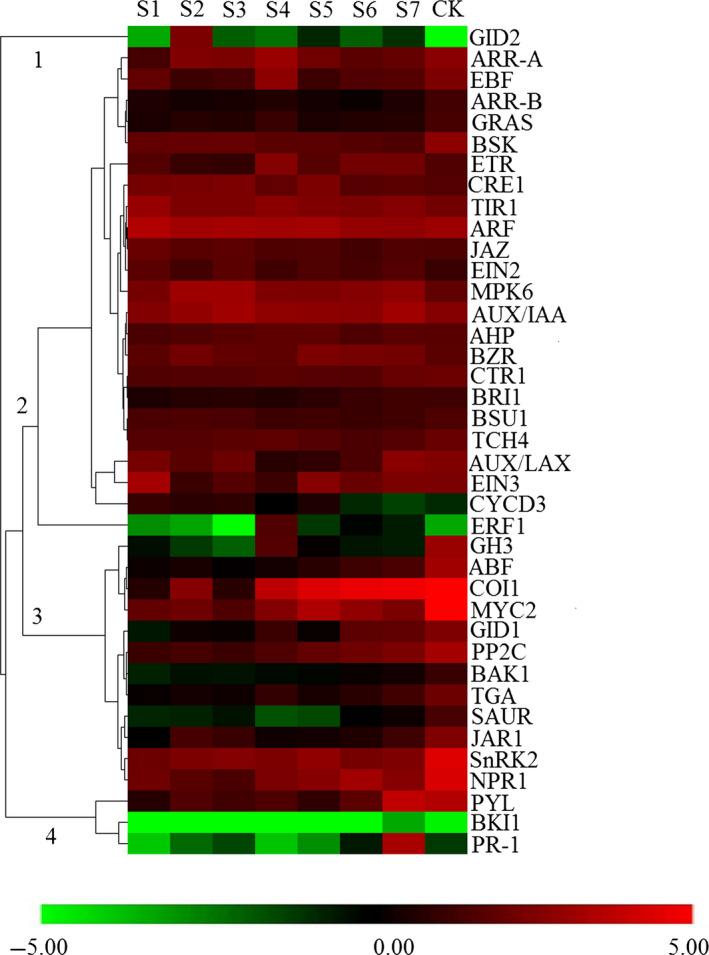
The average expression of plant hormone signalling‐related gene families at different growth stages. The number in the heat map represents different expression clusters. The colour scale represents log2‐transformed RPKM (reads per kilobase per million) values. Green indicates low expression, and red indicates high expression.

To comprehensively understand the potential roles of hormone signalling genes involved in moso bamboo shoot growth, we analysed gene expression patterns in a co‐expression network. The expression of hormone signalling genes could be divided into four groups (Figure 11). Cluster 1 had the high abundance of expression at S2 and then gradually decreased, until expression could not be detected in the mature period (CK). The majority of genes in cluster 2 were highly expressed in the early growth period (S1–S5) and lowly expressed in late growth period and CK (Figure 11). The highest expressed genes in the up‐regulated groups were *ARF* (auxin response factor), *AUX/IAA* and *MPK6*. The expression of cluster 3 and cluster 4 increased gradually with the development of the bamboo shoot. With the exception of the GH3 and SAUR families, all gene families involved in auxin signal transduction were generally more up‐regulated than down‐regulated genes (Tables S3 and S4). In terms of JA signalling pathways, 13 to 22 *MYC2‐like* genes were up‐regulated, while 9 to 18 genes were down‐regulated during shoot elongation. In JAZ family, the quantity of down‐regulated genes performed a gradual decreasing trend, while none of *JAZs* up‐regulated during shoot elongation.

**Figure 12:**
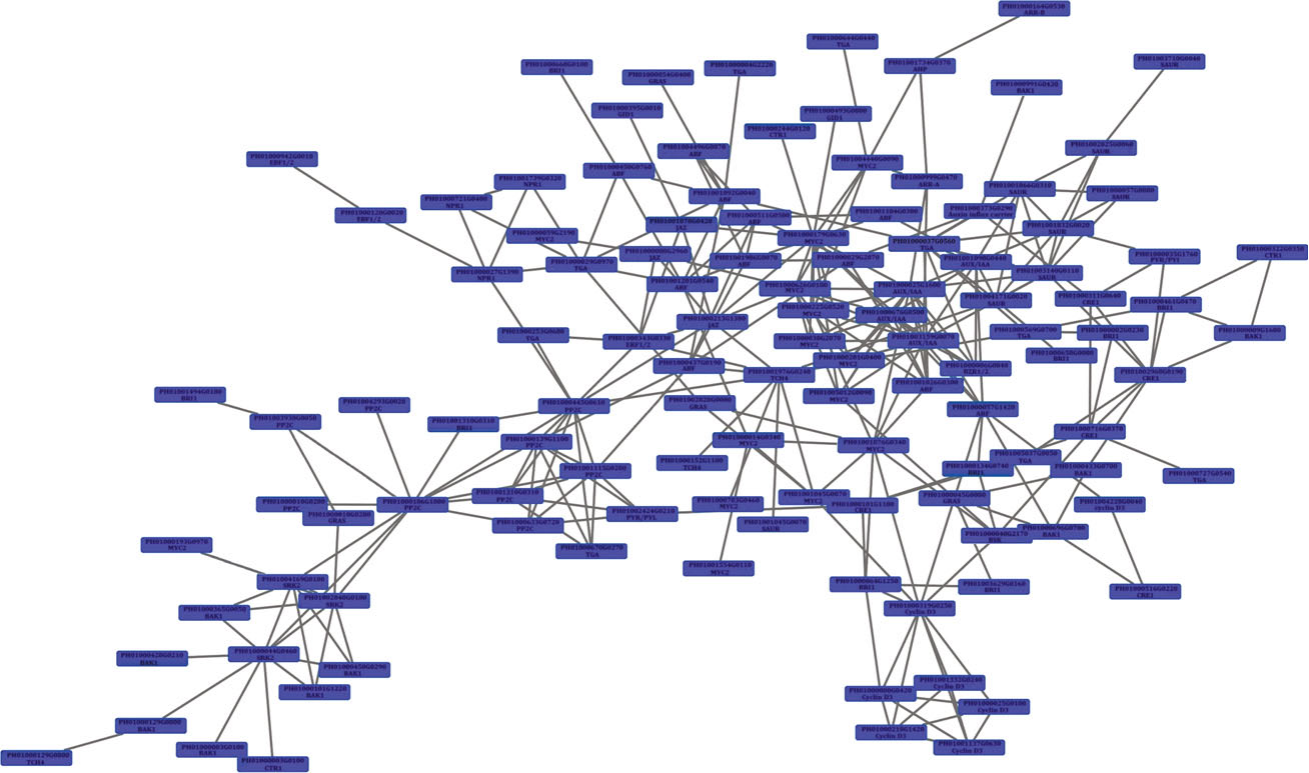
A co‐expression network of differentially expressed hormone signalling genes in moso bamboo.

To analyse the phytohormone interactions, a network was constructed comprising differentially expressed hormone signalling genes (Figure 12). Among the top fifteen most highly connected (DGEs ≥7) in the network (hub genes), five were involved in the auxin signalling pathway. Four of these genes were *AUX/IAA* family members. The auxin signalling pathway accounts for a higher quantity of hub genes than other hormone signalling pathways, indicating that auxin signal transduction, especially *AUX/IAA*, plays an important role in shoot growth (Table S5).

**Figure 13:**
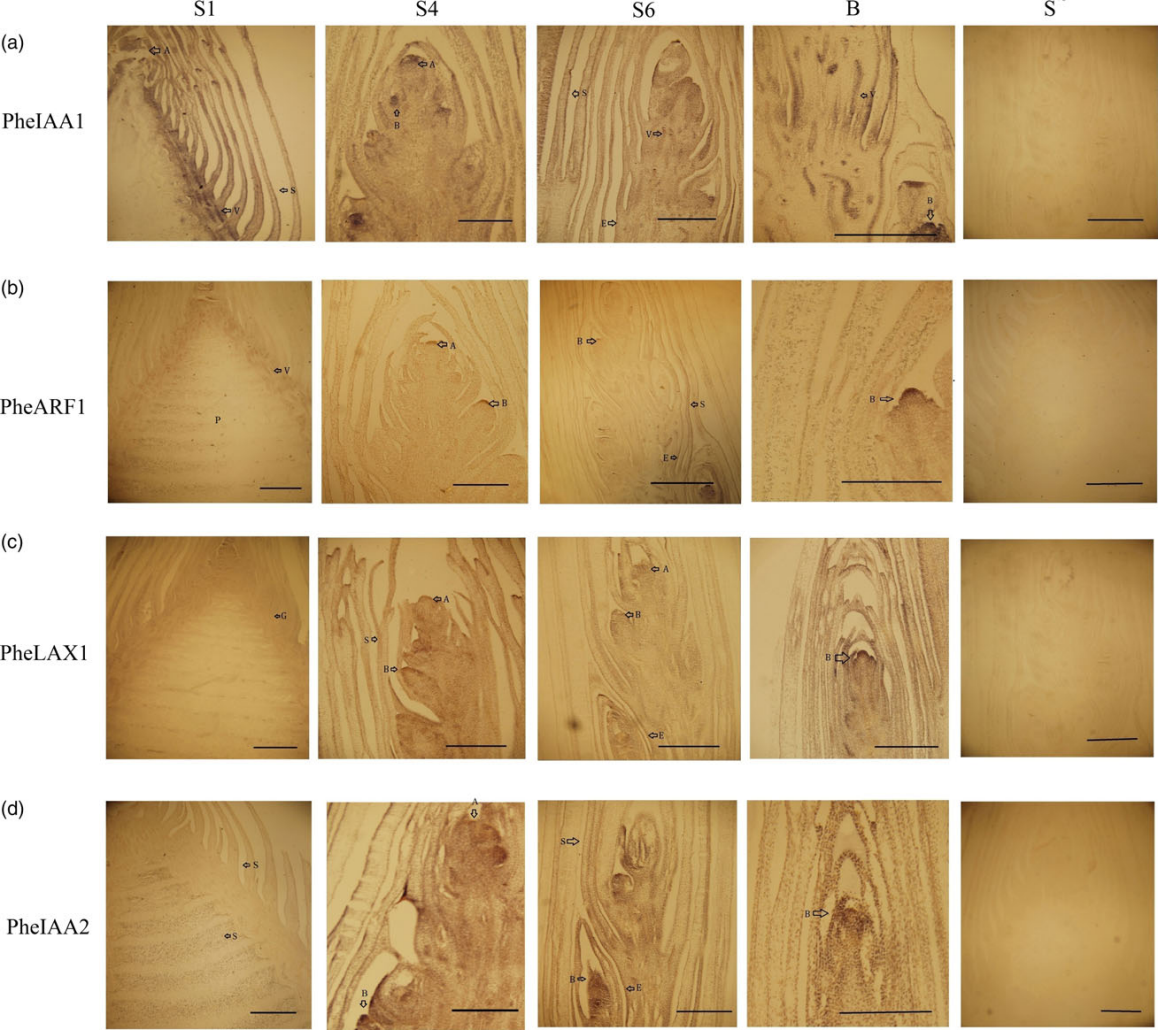
The expression of auxin signalling‐related genes in moso bamboo shoots at different growth stages. S1, S4, S6, B represents winter bamboo shoot, 3 m height shoot, 9 m height shoot and lateral buds, S represents the expression of the sense probe in *in situ* hybridization. Arrowheads point to transcript accumulation. A, B, E, G, P, S and V represent the apical bud, lateral bud, epidermis, ground tissues, pith, bamboo sheath and vascular bundle, respectively. Bars, 2 mm. (a) PH01000025G1600 (AUX/IAA), (b) PH01000046G0220 (ARF), (c) H01000373G0290 (AUX/LAX), (d) PH01001098G0440 (AUX/IAA).

Four auxin signalling genes that were significantly up‐regulated during bamboo shoot growth were further investigated with *in situ* hybridizations. In S1, *PheIAA1* had higher expression in ground tissues and bamboo sheaths than in pith meristems. In S4 and S6, *PheIAA1* was highly expressed in the lateral buds, the epidermis and in the vascular bundles (Figure 13a). *PheARF1* was expressed in the apical growth cones of the lateral buds, the apical buds and the epidermis, but nearly absent in bamboo sheaths and intercalary meristems (Figure 13b). All four genes were highly expressed in the apical growth cones of lateral buds and apical buds (Figure 13). Moreover, *PheLAX1*,* PheIAA1* and *PheIAA2* were also detected in the bamboo sheaths and epidermis (Figure 13a, c, and d). These results suggest that auxin signalling genes play various roles in the processes of bamboo shoot growth.

## Discussion

### The role of plant hormones involved in shoot growth

Here, we assessed the spatiotemporal variation of moso bamboo shoots during growth, revealing 10,344 DEGs that include relevant genes involved in plant hormone signalling. Following expression analysis indicated that the most of differentially expressed auxin signalling genes were up‐regulated during moso bamboo shoot growth, and they also contained much more connecting time than other hormone signalling genes in network.

Auxin exerts a rapid and specific regulation of auxin‐inducible genes at the transcriptional level. Increased *AUX/LAX1* activity reinforces the auxin‐dependent induction of certain cell wall remodelling enzymes, which can promote cell separation (Swarup *et al*., 2008). Seven *AUX/LAX1* members were differentially expressed in this study, with five being up‐regulated. Their high abundance may be essential for auxin polar transport and cell division during bamboo shoot growth. Many studies have demonstrated that SAUR proteins can promote cell elongation (Bai *et al*., 2016; Li *et al*., 2015). In the early growth period, *SAUR* gene families were lowly expressed, but as cell elongation replaced cell division in shoot growth, SAUR family expression increased. Thus, the high abundance of SAUR in the late growth period may be involved in cell elongation. In maize, the Aux/IAA protein RUM1 controls seminal and lateral root initiation. Histological analyses of *rum1* mutant roots revealed defects in the differentiation of pith cells around the xylem (Zhang *et al*., 2014). The high concentration of *PheIAA1* and *PheIAA2* in lateral buds, vascular bundles and intercalary meristems indicates that these genes may play vital roles in cell division and cell differentiation in these tissues. In *Arabidopsis*, the mutation of *iaa7* and *iaa3* resulted in the disappearance of apical dominance (Tian and Reed, 1999). We propose that the high concentration of *PheIAA1* and *PheIAA2* in apical bud may indicate a decisive function of *AUX/IAA* in maintaining apical dominance during moso bamboo shoot growth.

E2F transcription factors play decisive roles in cell size, which correlate with cell cycle regulation (Lammens *et al*., 2009; Sabelli and Larkins, 2009). The observed enhancement of transcript levels correlates with the regulatory factors of the cell cycle (Figure S4). Previous studies suggest that members of AP2 subfamily play important roles in auxin signalling in many model plants (Riechmann and Meyerowitz, 1998). Three to 17 AP2 TFs were differentially expressed with most being up‐regulated (Table S4), indicating that AP2 plays important roles in bamboo shoot growth via auxin signalling.

The JAZ family is a key repressor of JA signalling, which was generally down‐regulated during bamboo shoot growth. The degradation of JAZs by JA also has an impact on the activity of DELLA (the repressor of GA signalling), and thus, their low abundance may promote GA‐mediated developmental processes (Hou *et al*., 2013). We detected 13–22 *MYC2‐like* genes which are required for jasmonate biosynthesis and signalling, enhancing transcript levels during the shoot elongation period. Although JA signalling is related to pathogen defence and wound response, many studies suggested that MYC2 participates in plant development, lateral and adventitious root formation, and flowering time (Kazan and John, 2013). Their high abundance may facilitate bamboo growth through a direct or indirect effect.

In the co‐expression network, auxin signalling genes were considered ‘hub genes’ because of their high connectivity (Zhu *et al*., 2007). In the subnet, *PheIAA1* had 13 edges and was connected with cytokinin, BR (brassinosteroid) and JA (jasmonic acid) signalling genes. Except for PH01000179G0630, a MYC2 gene, all auxin signalling genes were up‐regulated, indicating the importance of *PheIAA1* involved in crosstalk between auxin signalling and other signals. A similar phenomenon was observed in *PheIAA2* and other AUX/IAA members. Four AUX/IAA genes were closely connected with BR signalling, and coincidently, the AUX/IAA‐mediated BR signal has been shown in *Arabidopsis* (Ayako *et al*., 2003). Cytokinins are also known to impact polar auxin transport through the modulation of auxin efflux carrier activity (five were up‐regulated in our data set; Su *et al*., 2011). Thus, it is likely that augmenting auxin efflux carrier activity would promote shoot elongation. Many new studies suggest that ARF families not only participate in auxin signalling, but also mediate cytokinin (Cheng *et al*., 2013; Kobayashi *et al*., 2012) and gibberellin signalling (Elena *et al*., 2015; Maaike *et al*., 2011). Our gene interaction network suggests a similar conclusion. For example, PH01000057G1420 can interact with AUX/IAA, cyclin D3 and BAK1 genes, which participate in cytokinin signalling and BR signalling, respectively. Taken together, we speculate that auxin signalling genes function as hub genes that trigger or coordinate other hormone signalling genes, thus playing an important role in moso bamboo shoot growth.

### Cell division and cell elongation are essential for shoot elongation

Numerous cell nuclei were found in both ground tissue and vascular tissue during the winter growth and early growth period, indicating the presence of meristematic tissue. As the shoot developed in the later period (8–12 m), the number of nuclei in the intercalary meristem declined, until only a small number of nuclei could be detected at 12 m. This trend coincided with expression of cell cycle‐related genes (cyclin A, cyclin B, cyclin D) and cell division (Figure S5). With the expression of these genes gradually decreasing from S6 to CK, cell division activity also decreased. In addition, numerous genes annotated as ‘oxidation–reduction process’, ‘monocarboxylic acid biosynthetic process’, ‘phenylpropanoid biosynthetic process’ and other biological processes were also found to be statistically significant with most being up‐regulated. This may be related to ongoing cell proliferation in the meristems (Gao *et al*., 2015; Sun *et al*., 2014). Furthermore, numerous mitochondria were present in the internode cells indicating the great energy requirements for cell proliferation during shoot growth.

Aquaporin genes take part in the absorption of water and cell elongation during auxin‐induced growth (Teale *et al*., 2006). Thus, a high abundance of aquaporins should be essential for cell differentiation during shoot growth in moso bamboo (Table S4). Nearly two‐thirds of kinesins were significantly up‐regulated during shoot growth. Previous studies have reported that the kinesin family has been shown to regulate the progression of cytokinesis (Hirokawa *et al*., 2009; Tanaka *et al*., 2004) and the formation of the cell plate (Michiko *et al*., 2015) in Arabidopsis. Thus the high expression of kinesins may be associated with organelle transport and chromosome segregation during bamboo shoot growth. Chromosome transmission fidelity protein and cyclin A associate with chromosome replication, all of which demonstrated a widespread up‐regulated tendency in this study (Table S4), indicating that parenchyma and fibre cells are undergoing mitosis during shoot elongation. In summary, the high abundance of cell division‐associated genes was essential for meristem cells to maintain vigorous growth in the winter growth period and early growth period. However, the growth pattern of the meristem in winter growth period and early growth period was quite different. In winter, meristem cell proliferation resulted in the primary thickening growth of the bamboo shoot, while the height of the bamboo shoot was almost unchanged (Wei *et al*., 2016). But in the early growth period, cell proliferation in meristem resulted in a fast elongation of internodes, eventually leading to longitudinal growth of bamboo shoot.

The cell wall expansion factors and enzymes, such as cellulose synthase‐like and xyloglucan endotransglucosylase/hydrolase, were highly expressed during the late growth period (Figure S5). In late period, the cell division ability of intercalary meristem declined and the continuous growth of the bamboo shoot were substituted by cell elongation (Cui *et al*., 2012). During auxin‐induced growth, their high abundance is essential for regulating cell wall plasticity through a loosening process (Paque *et al*., 2014).

### Spatiotemporal dynamic change of moso bamboo shoot

The biological characteristics of moso bamboo shoots have been thoroughly studied, although most research has focused on early shoot growth (Wei *et al*., 2016; Xu *et al*., 2008). Sequentially elongating internodes from the base to the top have been reported (Cui *et al*., 2012). A proteomics study demonstrated that many metabolic processes are involved in cell wall structure and the fast growth of bamboo (Cui *et al*., 2012). However, the spatiotemporal variation of moso bamboo shoots during the growth period is still unclear. In the winter growth period, when the whole bamboo shoot was covered by a thin layer of soil, the bamboo shoot completed primary thickening growth (Wei *et al*., 2016). By spring, the shoot emerged from the soil, while the lateral bud formed, indicating the beginning of the early growth period. The intercalary meristem of the internode from shoot tip grew vigorously in the early growth period, but declined in the late growth period. This trend was consistent in the middle internode, although growth declined much later (Cui *et al*., 2012). In addition, the number of alternative splicing events (AS) changed regularly during shoot growth. The AS frequency was the lowest in 1‐year‐old culm (CK) followed by the late growth period and early growth period, with winter bamboo shoot having the most (Li *et al*., 2016). Similarly, the early growth period had much more DGEs than in late growth period.

Plants are involved a complex crosstalk of plant hormones in which IAA, GA_3_, BR and ZR often act synergistically to promote plant growth and development (Maaike *et al*., 2011), while ABA is often regarded as an inhibitory factor (Nemhauser *et al*., 2006). Therefore, we calculated the ratio between growth promoting factors and the inhibitory factors to explore the relationship with shoot growth. Interestingly, this ratio increased in the top part of the meristem from stage S1 to S4 and decreased after (Figure 6f), which mirrored the trend in the quantity of DGEs (Figure 7a). However, the peak ratio appeared at S4, while the peak quantity of DGEs appeared at S3. Similarly, the ratio always coincided with the expression of cell cycle‐associated genes, indicating that endogenous hormones may affect the activities of cell cycle‐related genes (cyclin A, cyclin B, cyclin D and E2F) (Figure S5). According to these results, we propose a scenario for the bamboo shoot growth. The bamboo shoot completed the primary thickening growth in winter. When spring came, some environmental cues including moisture, temperature and fertile soil accelerate the concentration of IAA, GA3, BR and ZR, but repress the content of ABA. The concentration changes of endogenous hormone resulted in differential expression of hormone signalling genes in which auxin signalling played a key role. The expression of genes involved in cell division, cell cycle, metabolism and material transport could be triggered by hormone signalling pathways. Next, the meristems, which have ongoing cell proliferation, take up energy or nutrients, possibly supplied by the root system and mother bamboo rhizome. Simultaneous cell division and cell elongation affect internode elongation, while the former is predominant in the early growth period, while the latter is predominant in the late growth period, eventually leading to height increases in the bamboo shoot.

In conclusion, our research demonstrates the dynamic change of morphological anatomy and gene expression during shoot growth. This work also reveals how hormone signalling‐associated genes influence the moso bamboo shoot growth. This work provides key information for the further study of genes involved in the molecular mechanisms of shoot growth.

## Experimental procedures

### Sample preparation

Moso bamboo samples were collected in Lu'an City (E116°19′72″9; N31°23′30″), in the Anhui Province from January to August 2014. A laser altimeter, Nikon COOLSHOT AS, was used to measure the shoot height of five individuals at 8 A.M. every morning from April 2nd to June 20th. Seven different heights of shoot tips (winter bamboo shoot, 50, 100, 300, 600, 900 and 1200 cm) and culms after leaf expansion were selected in accordance with bamboo developmental stages labelled as S1, S2, S3, S4, S5, S6, S7 and CK, respectively. The sample collection and storage were consistent with previous work (Li *et al*., 2016).

### Paraffin sectioning

Shoot tips were dehydrated in an ethanol series, infiltrated with xylene, processed and embedded in paraffin sections. The embedded tissues were then sectioned at 15 μm thickness and stained with safranin and fast green for observation under an Olympus BX‐51 with a digital image acquisition system.

### Transmission electron microscopy

Tissues between the third and fourth bamboo joint were isolated from shoot tips under a dissecting microscope. Tissues were fixed in 2.5% (w/v) glutaraldehyde and rinsed thoroughly with 0.1 m phosphate buffer. Samples were post‐fixed with 1% osmium tetroxide, washed in 0.1 m phosphate buffer, dehydrated in an acetone series and then embedded in Spurr's resin. Thin sections were cut with an LEICAUC6I microtome and examined with a JEM‐123O transmission electron microscope.

### Endogenous hormone measurement

For endogenous hormone measurements, each bamboo shoot was divided into basal (B), middle (M) and top parts (T) by height using an equal division method. The first internode and bamboo joint were collected for B, while the middle internode and bamboo joint on its bottom from the middle part were collected. For T, the tissues above the fifth bamboo joint (containing the fifth bamboo joint) were separated and collected. Extraction and purification of endogenous hormones, indole‐3‐acetic acid (IAA), gibberellic acid (GA_3_), abscisic acid (ABA), brassinosteroids (BR) and cytokinin zeatin riboside (ZR) were performed as previously (Wang *et al*., 2009). ELISA was used for the estimation of hormone levels, with three biological replicates for each set of experiments.

### cDNA library construction

Total RNA from each sample was isolated using Trizol (Invitrogen, Carlsbad, California, USA). RNA quality was tested on an agarose gel, NanoDrop8000 spectrophotometer (NanoDrop, Thermo Scientific, Waltham, Massachusetts, USA) and an Agilent 2100 Bioanalyzer, USA. The cDNA libraries from eight different samples were constructed using Illumina's kit (Illumina, San Diego, CA). The library was sequenced with 101 bp paired‐end reads using an Illumina HiSeq™ 2000 at Macrogen in Shenzhen, China.

### Reads mapping to reference genome and genes

The raw RNA‐seq in FASTQ format with a quality score of Phred ≥20 was indexed, trimmed and aligned. The clean reads were then aligned to the moso bamboo genome and gene set obtained from the National Center for Genome Research (http://www.ncgr.ac.cn/bamboo) using TopHat. Cufflinks was used to measure the relative abundance of transcripts with the FPKM method (fragments per feature kilobase per million reads; Ali *et al*., 2008). The identification of significant DGE models between different samples was performed with Cuffdiff (Trapnell *et al*., 2010). Differentially expressed transcripts (FDR value ≤0.05 and ≥1 fold change) were annotated and categorized automatically with Blast2go GO (Gene Ontology, http://www.blast2go.com/b2ghome.) To analyse phytohormone interactions, a network of all differentially expressed phytohormone‐related genes was constructed using WGCNA and visualized using Cytoscape (v.3.1.0).

### Quantitative real‐time PCR verification

To estimate the validity of the transcriptome sequencing, six genes were selected randomly and analysed with qRT‐PCR. qRT‐PCR of shoots at different growth stages was performed separately using culms after leaf expansion as the control. Total RNA was extracted using Trizol (Invitrogen), 2 mg of total RNA was reverse‐transcribed to the first‐stand cDNA using M‐MLVRT (Promega). The primers were designed using Primer 3 software (http://www.genome.wi.mit.edu/cgi-bin/primer/primer3.cgi) (Table S1). *TIP41* (tonoplast intrinsic protein 41) was used as an internal control (Fan *et al*., 2013; Peng *et al*., 2013a). PCR was conducted using a fluorescent intercalating dye in a Light Cycler 480 SYBR Green I Master Mix (Roche, Mannheim, Germany; Roche). Cycling conditions were 95 °C for the initial 10 min, followed by 40 cycles of (10 s at 95 °C, 10 s at 60 °C and 20 s at 72 °C). All reactions were performed in triplicate, both technical and biological.

### *In situ* hybridization

Moso bamboo shoots were fixed in 4% paraformaldehyde for 24 h, dehydrated, embedded in paraffin, and 10 μm thick sections were prepared with a microtome (Leica, Germany) and mounted on slides. The slides were dehydrated and baked, followed by dewaxing with dimethylbenzene (Hord *et al*., 2006). *In situ* probes (antisense and sense) of PH01000025G1600 (GenBank: AOT28187, *PheIAA1*), PH01000046G0220 (GenBank: AOT28201, *PheARF1*), H01000373G0290 (GenBank: AOT28203, *PheLAX1*) and PH01001098G0440 (GenBank: AOT28188, *PheIAA2*) were PCR amplified using gene‐specific primers with T7 and SP6 RNA polymerase‐binding sites. Antidigoxigenin antibodies coupled with NBT/BCIP solution were used to detect hybridization signals.

## Author contributions

Long Li performed bioinformatics analyses, *in situ* hybridization experiments and drafted the manuscript. Zhanchao Cheng and Yanjun Ma co‐performed RNA extractions, qRT‐PCR, endogenous hormone measurement and anatomical experiments. Qingsong Bai and Xiangyu Li assisted in bioinformatics analyses. Zhihua Chao and Zhongneng Wu helped in sample collection. Jian Gao designed the experiments and conceived the project, provided overall supervision of the study and revised the manuscript. All authors have read and approved the final manuscript.

## Conflict of interest

All the authors have declared no conflict of interest.

## Supporting information


**Figure S1** Dynamic changes of the shoot tip during the growth period. Bars, 2 mm. Sampling times are shown under each section.
**Figure S2** The quality of the eight transcriptomes.
**Figure S3** K‐means clustering of differentially expressed genes. Yellow represents genes that have high expression and blue indicates low expression.
**Figure S4** Functional categorization of differentially expressed genes.
**Figure S5** Heat map generated by average expression of families associated with shoot growth.

**Table S1** Selected genes and primers used in qRT‐PCR analysis.
**Table S2** List of genes that are differentially expressed in developing shoot.
**Table S3** The down‐regulated genes involved in shoot growth.
**Table S4** The up‐regulated genes involved in shoot growth.
**Table S5** The number of interaction genes.
