# Supplementary material for: The association of hormone signalling genes, transcription and changes in shoot anatomy during moso bamboo growth
Source: Plant Biotechnol J. 2017 Jun 9;16(1):72–85. doi: 10.1111/pbi.12750 (PMC5785349; doi:10.1111/pbi.12750)

Supplementary Fig. S1. Dynamic changes of the shoot tip during the whole growth period. Bars, 2mm. Sampling times were shown under each sections.

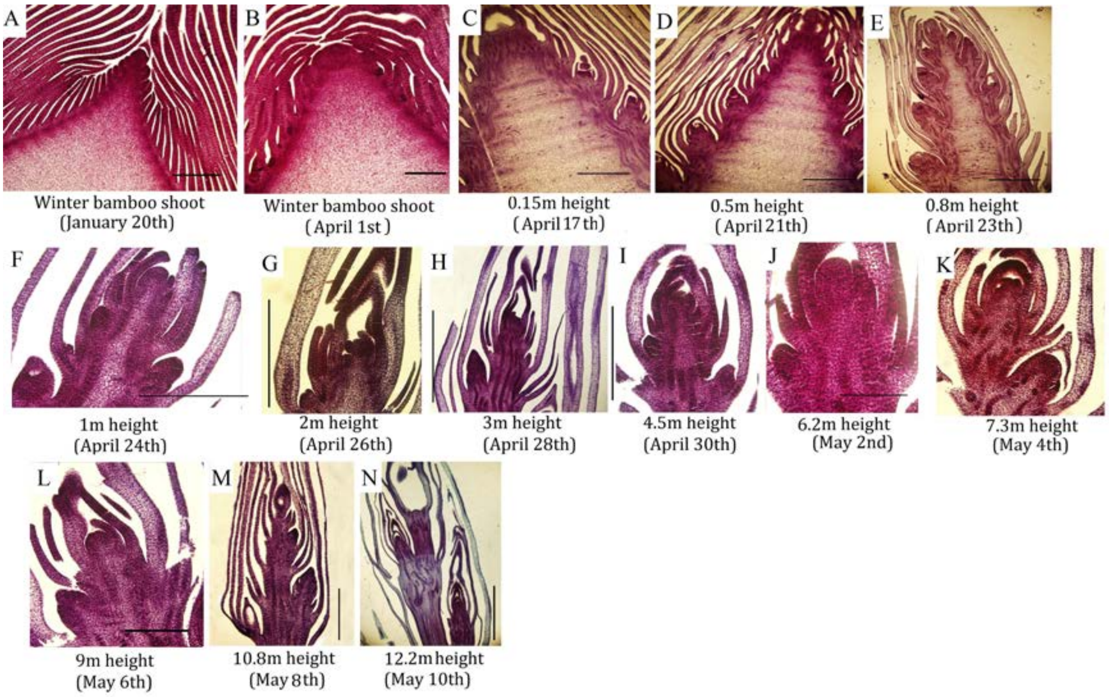

Supplementary Fig. S2. The qualities of the eight transcriptomes.

## Total reads of eight samples

| Sample | Total Bases | Read Count | GC(%) | Q20(%) | Q30(%) |
|---|---|---|---|---|---|
| 1 | 5625242470 | 55695470 | 51.1 | 96.2 | 91.1 |
| 2 | 5941728798 | 58828998 | 49.2 | 96.5 | 91.9 |
| 3 | 5627801002 | 55720802 | 50.5 | 96.4 | 91.6 |
| 4 | 5932167936 | 58734336 | 50.5 | 96.3 | 91.5 |
| 5 | 5657219070 | 56012070 | 50.9 | 96.3 | 91.4 |
| 6 | 5106518388 | 50559588 | 50.0 | 96.5 | 91.7 |
| 7 | 5049177860 | 49991860 | 50.7 | 96.3 | 91.4 |
| 8 | 5329125418 | 52763618 | 51.8 | 95.9 | 90.8 |

## Clean reads of eight samples

| Sample | Total Bases | Read Count | GC(%) | Q20(%) | Q30(%) |
|---|---|---|---|---|---|
| 1 | 4857367952 | 48092752 | 51.1 | 99.4 | 96.2 |
| 2 | 5208939660 | 51573660 | 49.5 | 99.4 | 96.2 |
| 3 | 4903211044 | 48546644 | 50.3 | 99.5 | 96.2 |
| 4 | 5162673580 | 51115580 | 50.4 | 99.4 | 96.1 |
| 5 | 4903720690 | 48551690 | 50.5 | 99.4 | 96.2 |
| 6 | 4465859632 | 44216432 | 50.8 | 99.3 | 96.2 |
| 7 | 4379119014 | 43357614 | 50.7 | 99.4 | 96.2 |
| 8 | 4545418342 | 45004142 | 51.2 | 99.4 | 96.2 |

Supplementary Fig. S3. K-means clustering of differentially expressed genes. Yellow represents genes that have high expression and blue indicates low expression.

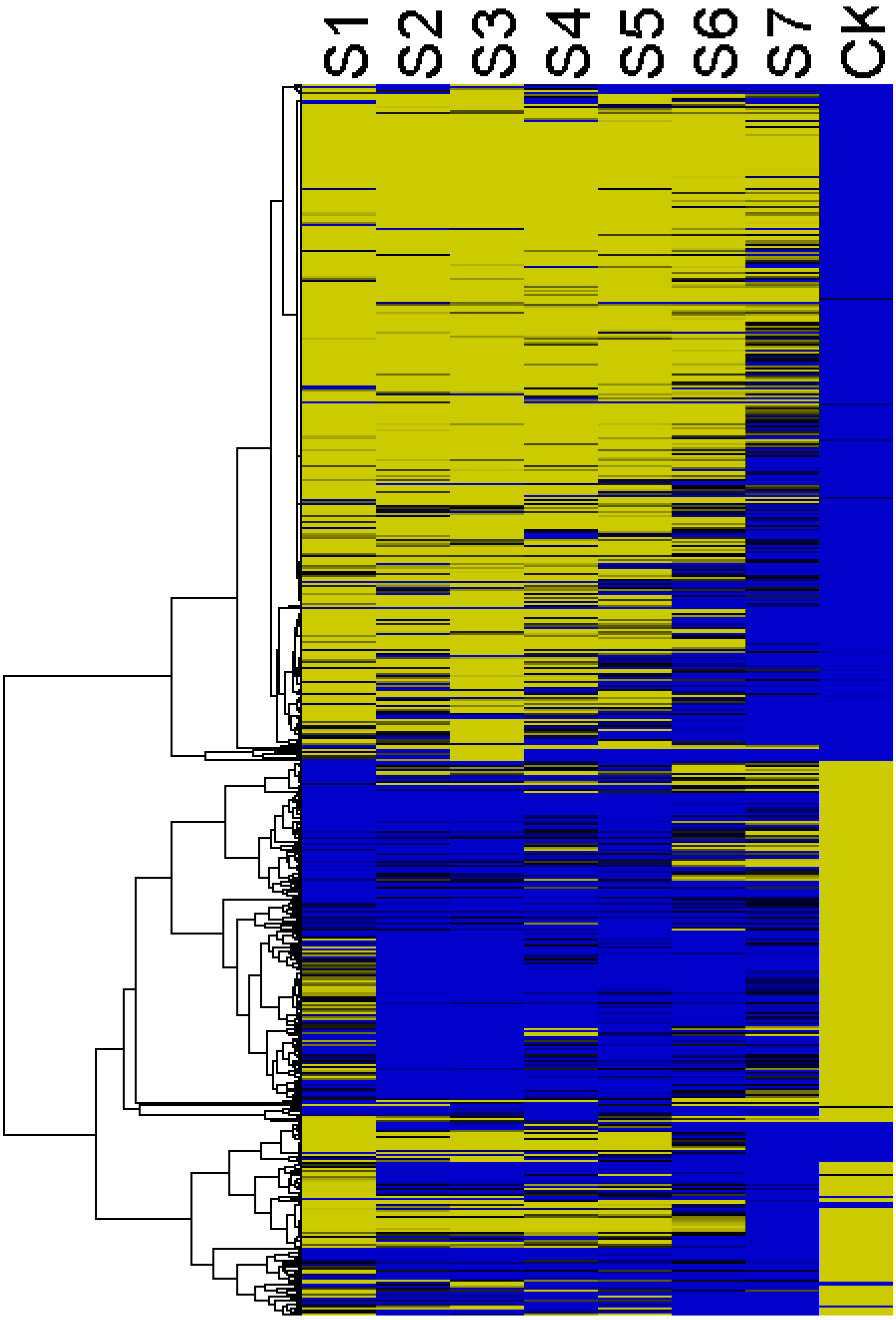

Supplementary Fig. S4. Functional categorization of differentially expressed genes.

| GO ID | GO Term | Type | FDR | Single Test P-Value | # in Test Group | # in Reference Group | # non Annot Test | # non Annot Reference Group | Over/ Under |
|---|---|---|---|---|---|---|---|---|---|
| GO:0016023 | cytoplasmic membrane-bounded vesicle | C | 4.3E-35 | 1.4E-38 | 1058 | 4368 | 2444 | 17287 | OVER |
| GO:0003700 | sequence-specific DNA binding transcription factor activity | F | 6.8E-10 | 6.1E-13 | 200 | 682 | 3302 | 20973 | OVER |
| GO:0005576 | extracellular region | C | 1.2E-8 | 1.6E-11 | 125 | 373 | 3377 | 21282 | OVER |
| GO:0009755 | hormone-mediated signaling pathway | P | 2.3E-8 | 3.8E-11 | 263 | 1027 | 3239 | 20628 | OVER |
| GO:0020037 | heme binding | F | 1.9E-7 | 5.4E-10 | 89 | 245 | 3413 | 21410 | OVER |
| GO:0004675 | transmembrane receptor protein serine/threonine kinase activity | F | 1.9E-7 | 6.6E-10 | 164 | 579 | 3338 | 21076 | OVER |
| GO:0007178 | transmembrane receptor protein serine/threonine kinase signaling pathway | P | 1.9E-7 | 6.6E-10 | 164 | 579 | 3338 | 21076 | OVER |
| GO:0001653 | peptide receptor activity | F | 1.9E-7 | 6.6E-10 | 164 | 579 | 3338 | 21076 | OVER |
| GO:0055114 | oxidation-reduction process | P | 2.8E-7 | 1.0E-9 | 356 | 1548 | 3146 | 20107 | OVER |
| GO:0046777 | protein autophosphorylation | P | 6.7E-7 | 2.6E-9 | 180 | 669 | 3322 | 20986 | OVER |
| GO:0031625 | ubiquitin protein ligase binding | F | 1.5E-6 | 6.1E-9 | 170 | 630 | 3332 | 21025 | OVER |
| GO:0009506 | plasmodesma | C | 1.8E-6 | 8.7E-9 | 205 | 805 | 3297 | 20850 | OVER |
| GO:0005506 | iron ion binding | F | 4.2E-5 | 2.5E-7 | 80 | 248 | 3422 | 21407 | OVER |
| GO:0004601 | peroxidase activity | F | 4.3E-5 | 2.7E-7 | 51 | 128 | 3451 | 21527 | OVER |
| GO:0072330 | monocarboxylic acid biosynthetic process | P | 6.2E-5 | 4.2E-7 | 108 | 379 | 3394 | 21276 | OVER |
| GO:0009611 | response to wounding | P | 1.0E-4 | 7.5E-7 | 33 | 66 | 3469 | 21589 | OVER |
| GO:0009523 | photosystem II | C | 2.5E-4 | 2.0E-6 | 26 | 46 | 3476 | 21609 | OVER |
| GO:0071554 | cell wall organization or biogenesis | P | 1.3E-3 | 1.1E-5 | 142 | 582 | 3360 | 21073 | OVER |
| GO:0051119 | sugar transmembrane transporter activity | F | 2.2E-3 | 2.0E-5 | 30 | 69 | 3472 | 21586 | OVER |
| GO:0008422 | beta-glucosidase activity | F | 3.1E-3 | 2.8E-5 | 15 | 20 | 3487 | 21635 | OVER |
| GO:0016709 | oxidoreductase activity, acting on paired donors, with incorporation or reduction of molecular oxygen, NAD(P)H as one donor, and incorporation of one atom of oxygen | F | 3.6E-3 | 3.4E-5 | 36 | 95 | 3466 | 21560 | OVER |
| GO:0005887 | integral component of plasma membrane | C | 3.8E-3 | 3.7E-5 | 42 | 120 | 3460 | 21535 | OVER |
| GO:0035428 | hexose transmembrane transport | P | 3.8E-3 | 3.8E-5 | 19 | 33 | 3483 | 21622 | OVER |
| GO:0046323 | glucose import | P | 3.8E-3 | 3.8E-5 | 19 | 33 | 3483 | 21622 | OVER |
| GO:0009679 | hexose:proton symporter activity | F | 4.2E-3 | 4.5E-5 | 6 | 1 | 3496 | 21654 | OVER |
| GO:0009538 | photosystem I reaction center | C | 4.2E-3 | 4.5E-5 | 6 | 1 | 3496 | 21654 | OVER |
| GO:0005355 | glucose transmembrane transporter activity | F | 4.6E-3 | 5.2E-5 | 19 | 34 | 3483 | 21621 | OVER |
| GO:0043565 | sequence-specific DNA binding | F | 5.4E-3 | 6.4E-5 | 94 | 365 | 3408 | 21290 | OVER |
| GO:0009834 | plant-type secondary cell wall biogenesis | P | 5.4E-3 | 6.5E-5 | 22 | 45 | 3480 | 21610 | OVER |
| GO:0009699 | phenylpropanoid biosynthetic process | P | 5.4E-3 | 6.5E-5 | 22 | 45 | 3480 | 21610 | OVER |

Supplementary Fig. S5. Heat map generated by average expression of families associated with shoot growth.

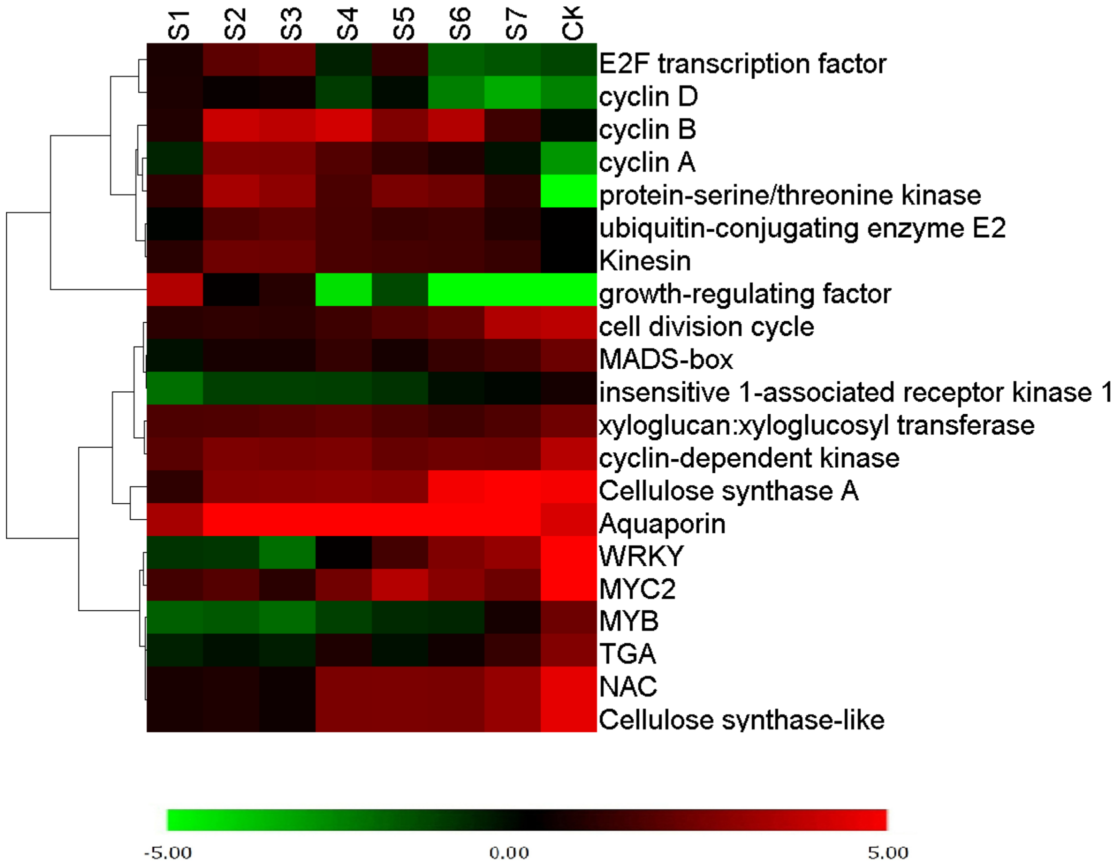

Supplement: Supplementary file 1 — Figure S1 Dynamic changes of the shoot tip during the growth period. Bars, 2 mm. Sampling times are shown under each section. Figure S2 The quality of the eight transcriptomes. Figure S3 K‐means clustering of differentially expressed genes. Yellow represents genes that have high expression and blue indicates low expression. Figure S4 Functional categorization of differentially expressed genes. Figure S5 Heat map generated by average expression of families associated with shoot growth. [file PBI-16-72-s001.pdf]
